# Supplementary figures and images for: Efficacy of laparotomy sponges to reduce bacterial contamination using an in vitro gastrointestinal surgery model
Source: PLoS One. 2022 Apr 29;17(4):e0267293. doi: 10.1371/journal.pone.0267293 (PMC9053784; doi:10.1371/journal.pone.0267293)

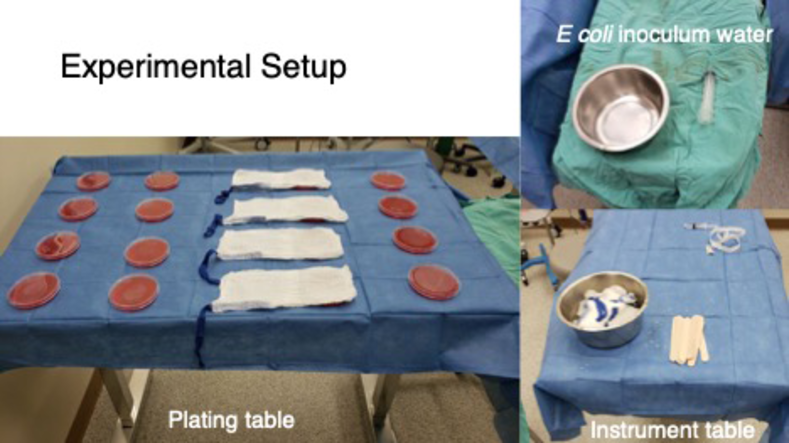

Supplement: S1 Fig — (TIF) [file pone.0267293.s001.tif]

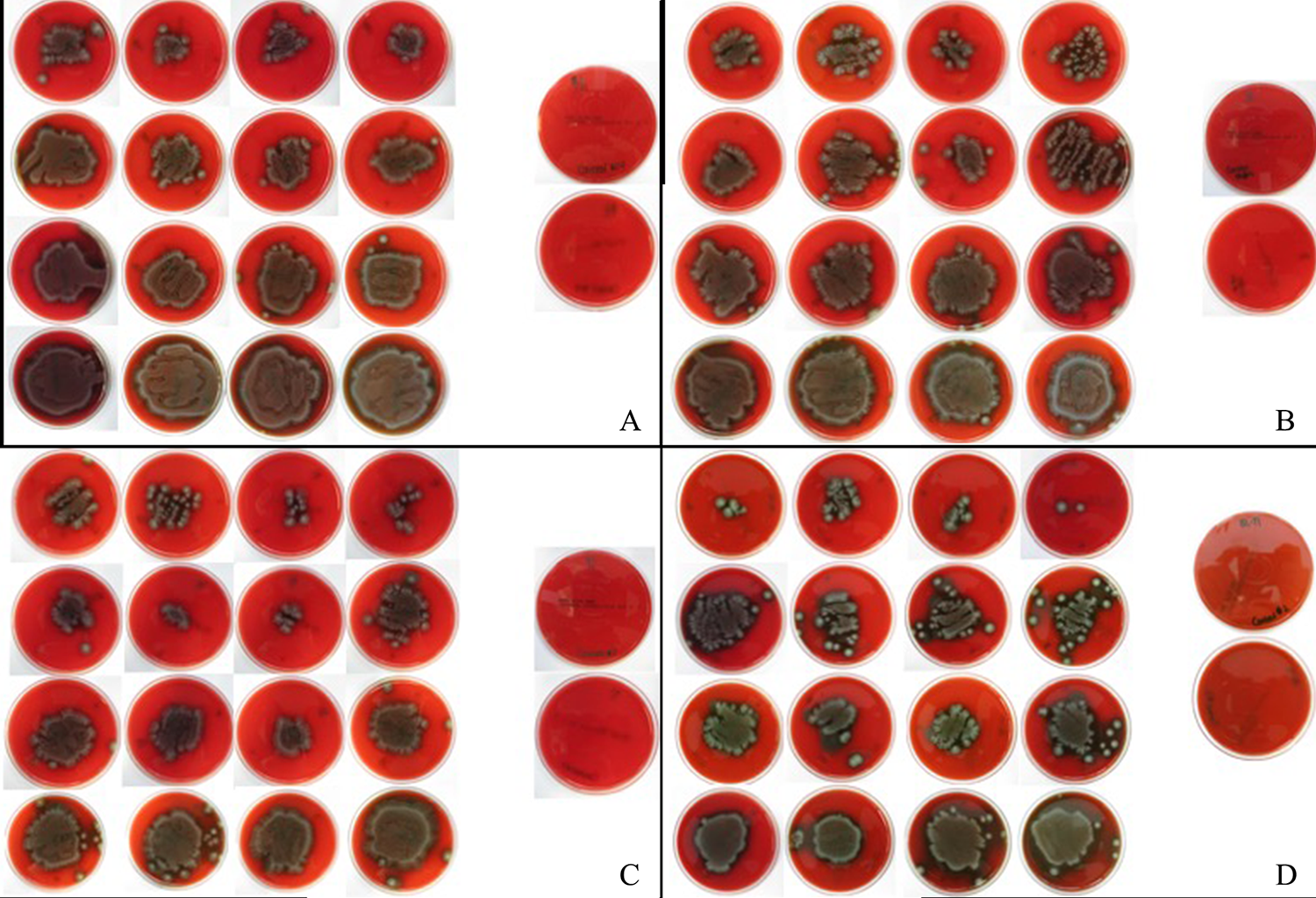

Supplement: S2 Fig — A–Trial 1 for 2 Layers. B–Trial 1 for 4 Layers. C–Trial 1 for 6 Layers. D–Trial 1 for 8 Layers. (TIF) [file pone.0267293.s002.tif]

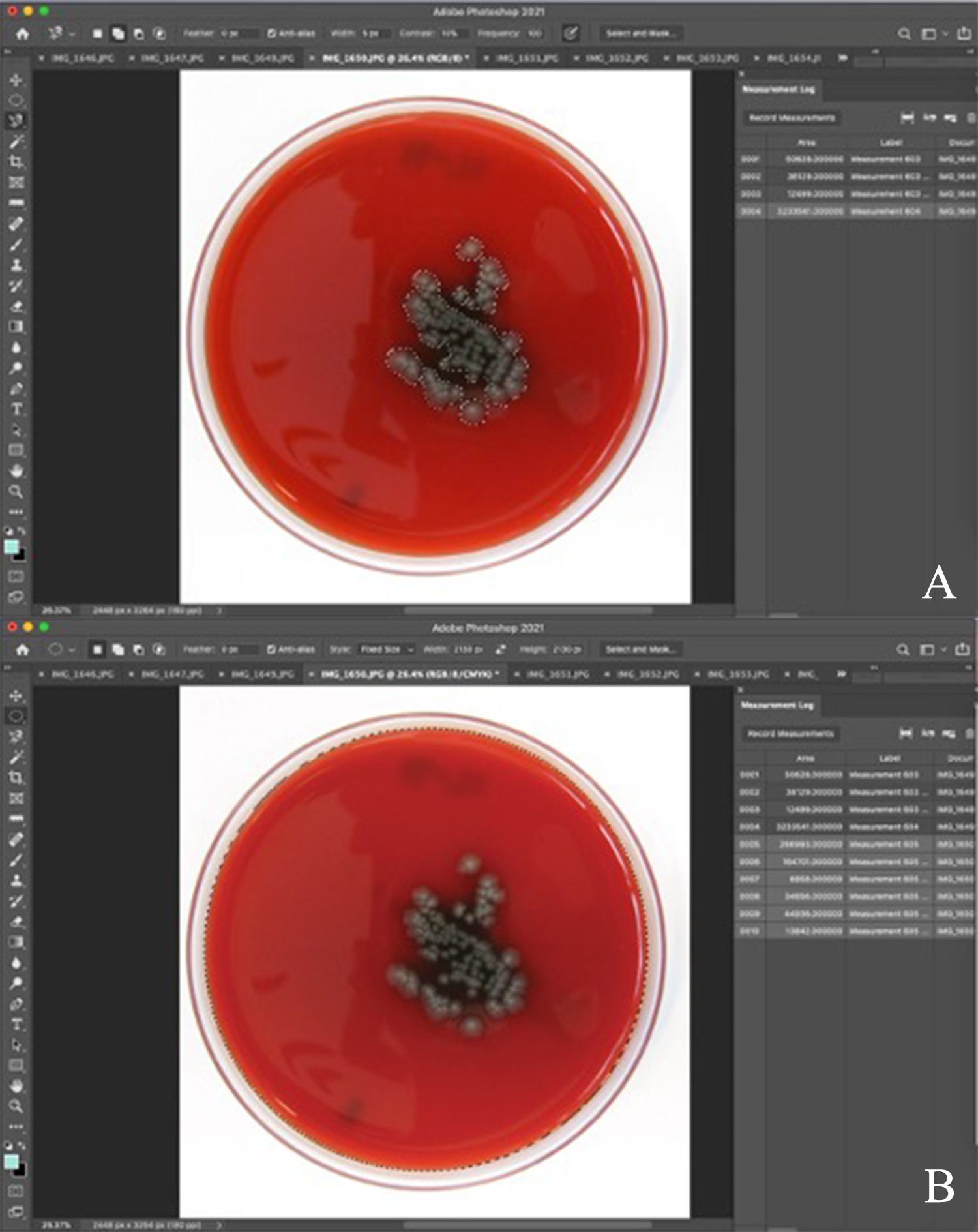

Supplement: S3 Fig — A–infected area in pixels, and B–entire blood agar medium on plate in pixels. (TIF) [file pone.0267293.s003.tif]
