# Supplementary material for: Efficacy of laparotomy sponges to reduce bacterial contamination using an in vitro gastrointestinal surgery model
Source: PLoS One. 2022 Apr 29;17(4):e0267293. doi: 10.1371/journal.pone.0267293 (PMC9053784; doi:10.1371/journal.pone.0267293)
Supplement: Attachment — Submitted filename: Response to Reviewers.docx [file pone.0267293.s004.docx]

To the academic editor and reviewers,

Thank you for your review and evaluation of my manuscript. Please find my responses to all comments and requested changes below:

Journal Requirements:

When submitting your revision, we need you to address these additional requirements.

1. Please ensure that your manuscript meets PLOS ONE's style requirements, including those for file naming. The PLOS ONE style templates can be found at 
<https://journals.plos.org/plosone/s/file?id=wjVg/PLOSOne_formatting_sample_main_body.pdf> and 
<https://journals.plos.org/plosone/s/file?id=ba62/PLOSOne_formatting_sample_title_authors_affiliations.pdf>

The necessary changes have been made to the manuscript to meet the PLOS ONE style requirements.

2. Please review your reference list to ensure that it is complete and correct. If you have cited papers that have been retracted, please include the rationale for doing so in the manuscript text, or remove these references and replace them with relevant current references. Any changes to the reference list should be mentioned in the rebuttal letter that accompanies your revised manuscript. If you need to cite a retracted article, indicate the article’s retracted status in the References list and also include a citation and full reference for the retraction notice.

The authors have reviewed all cited references and no retracted articles have been cited.

While revising your submission, please upload your figure files to the Preflight Analysis and Conversion Engine (PACE) digital diagnostic tool, <https://pacev2.apexcovantage.com/>. PACE helps ensure that figures meet PLOS requirements. To use PACE, you must first register as a user. Registration is free. Then, login and navigate to the UPLOAD tab, where you will find detailed instructions on how to use the tool. If you encounter any issues or have any questions when using PACE, please email PLOS at [figures@plos.org](mailto:figures@plos.org). Please note that Supporting Information files do not need this step.

All figures have been uploaded and evaluated by PACE.

______________________________________________________________________________

Reviewer #1: This is the first in vitro study assessing the efficacy of laparotomy sponges of various layer-densities tested at different contamination volumes and during different exposure times in the reduction of E. coli contamination.
The study provides novel information for veterinarians or surgeons who perform gastrointestinal surgery, to which authors recommed to use laparotomy songes with a minimum of 6 layers.
My only suggestion before publication is to reduce the introduction and to move figure legends and tables at the end of the manuscript.

Thank you, Reviewer #1. The introduction has been reduced in length. The authors would like to comply with this reviewer’s request to move figure legends and tables to the end of the manuscript. However, the PLOS ONE manuscript style requirements for submission require that figure captions are inserted “immediately following the paragraph in which the figure is first cited,” and the same applies for all tables.

Reviewer #2: Minimizing the risk of bacterial contamination in laparotomies is extremely important, due to the risk of peritonitis and patient death. The primary purpose of the present study was to evaluate the efficacy of laparotomy sponges in reducing bacterial contamination in an in vitro model and to evaluate whether bacterial contamination is dependent upon layer-density of laparotomy sponges or volume of contaminant.
But some points need to be clarified.

1. Methods:
- Experimental procedure – page 13 – lines 144-148: “…sponges were soaked, squeezed out manually, and each folded into the layer-density being evaluated per trial. For 2 layers, 1 sponge was folded in half once. For 4 layers, 1 sponge was folded in half twice. For 6 layers, 1 sponge was folded in half twice and a second sponge was folded in half once and these were stacked. For 8 layers, 2 sponges were each folded in half twice and these were stacked.” It was not clear whether only one side of the sponges or both sides were seeded onto the agar Petry plates.

Thank you for your comment and bringing this to the authors’ attention. I have clarified these points in the revised manuscript lines 205-209. Only one surface of the folded and/or stacked laparotomy sponges is in contact with the blood agar to model contact how a laparotomy sponge would contact abdominal viscera during surgical procedures.

2. Results:
- Page 17 - Table 1 - put the meaning of SE.

The meaning of SE has been added to the table legend.
